# Occurrence and treatment of peripheral nerve injuries after cosmetic surgeries

**DOI:** 10.3389/fneur.2023.1258759

**Published:** 2023-10-20

**Authors:** Qiang Chen, Pengfei Li, QingFang Zhao, Tian Tu, Hui Lu, Wei Zhang

**Affiliations:** ^1^Department of Hand & Reconstructive Surgery, Center for Plastic & Reconstructive Surgery, Zhejiang Provincial People’s Hospital, Affiliated People’s Hospital, Hangzhou Medical College, Hangzhou, Zhejiang, China; ^2^Department of Plastic and Aesthetic Center, First Affiliated Hospital, College of Medicine, Zhejiang University, Hangzhou, Zhejiang, China; ^3^Department of Plastic Surgery, First Affiliated Hospital, College of Medicine, Zhejiang University, Hangzhou, Zhejiang, China; ^4^Department of Orthopaedics, First Affiliated Hospital, College of Medicine, Zhejiang University, Hangzhou, Zhejiang, China; ^5^Department of Gastrointestinal Surgery, The Second Affiliated Hospital of Zhejiang Chinese Medical University, Hangzhou, China

**Keywords:** nerve injuries, peripheral nerves, plastic surgeries, cosmetic surgeries, treatment algorithm, occurrence

## Abstract

Although non-invasive and minimally invasive aesthetic procedures increasingly dominate the cosmetic market, traditional plastic surgery remains the most effective improvement method. One of the most common complications in plastic surgery, peripheral nerve injuries, though has a low incidence but intrigued plastic surgeons globally. In this article, a narrative review was conducted using several databases (PubMed, EMBASE, Scopus, and Web of Science) to identify peripheral nerve injuries following cosmetic surgeries such as blepharoplasty, rhinoplasty, rhytidectomy, breast surgeries, and abdominoplasty. Surgery-related nerve injuries were discussed, respectively. Despite the low incidence, cosmetic plastic surgeries can cause iatrogenic peripheral nerve injuries that require special attention. The postoperative algorithm approaches can be effective, but the waiting and treatment processes can be long and painful. Preventive measures are undoubtedly more effective than postoperative remedies. The best means of preventing disease is having a good understanding of anatomy and conducting a careful dissection.

## Introduction

Plastic surgeons are cognizant that non-invasive and minimally invasive aesthetic procedures with limited complications and downtime are gradually capturing a significant share of the market (1). Traditional cosmetic plastic surgeries are still indispensable due to their high effectiveness (2). Despite the low occurrence, peripheral nerve injuries are one of the common complications that can be a complex problem with various causes (3, 4). Besides causing patients significant anxiety, they also frustrate practitioners. Chronic pain, hyperesthesia, hypoesthesia, and numbness are common symptoms of peripheral nerve injury that must be considered seriously to distinguish them from infectious, neoplastic, and wound-related causes that can be prevented with antibiotics and proper tissue handling (5, 6).

**Figure 1:**
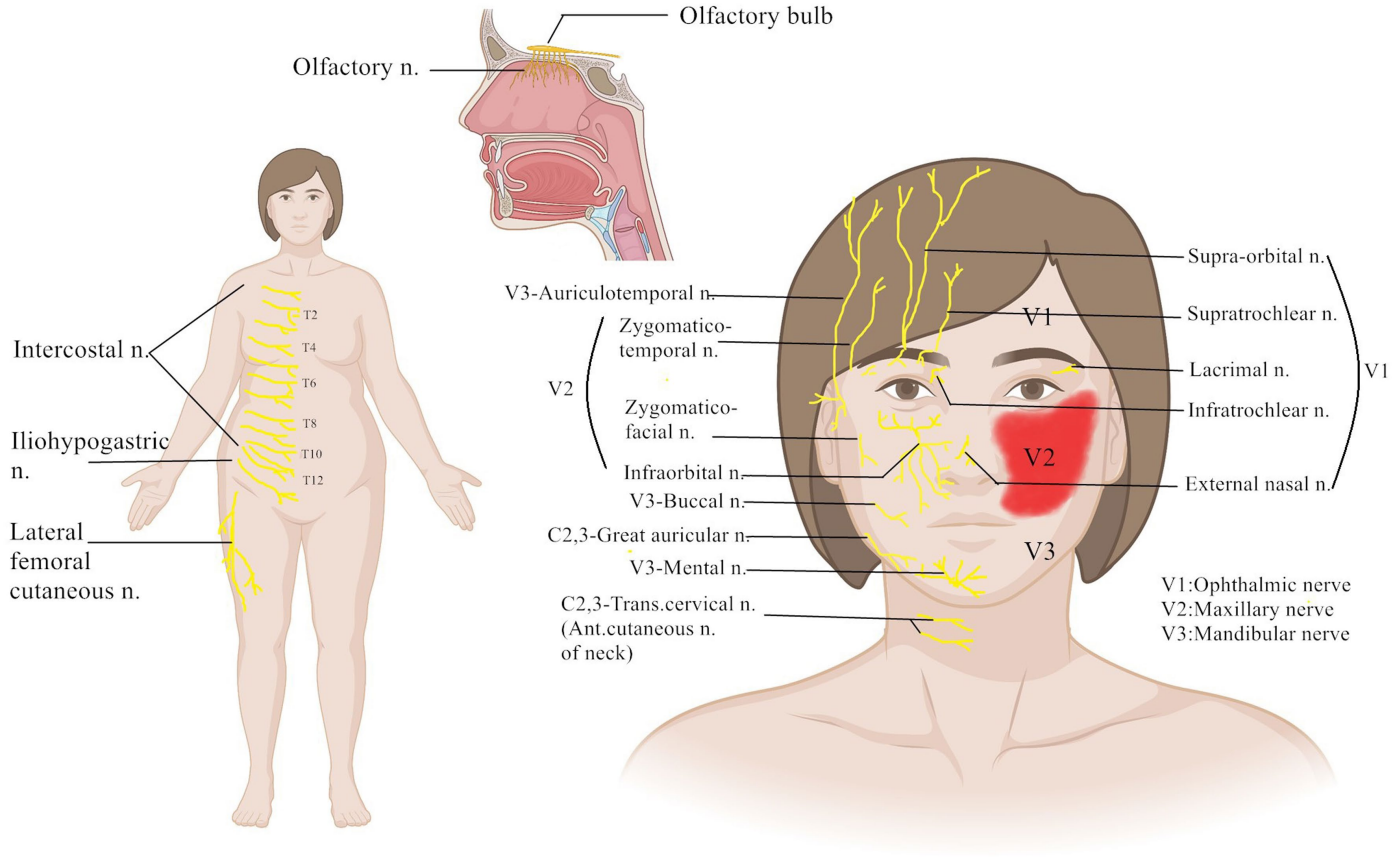
Distribution of peripheral nerves associated with common cosmetic surgery.

The purpose of this article was to comprehensively review the literature on peripheral nerve injuries associated with cosmetic surgery (as shown in Figure 1). The specific nerve injuries that occur following cosmetic surgery, such as faciocervical, breast, and abdominal procedures, will be discussed, respectively. Injuries of this type are also subject to potential treatments.

## Methods

### Literature search strategy

**Figure 2:**
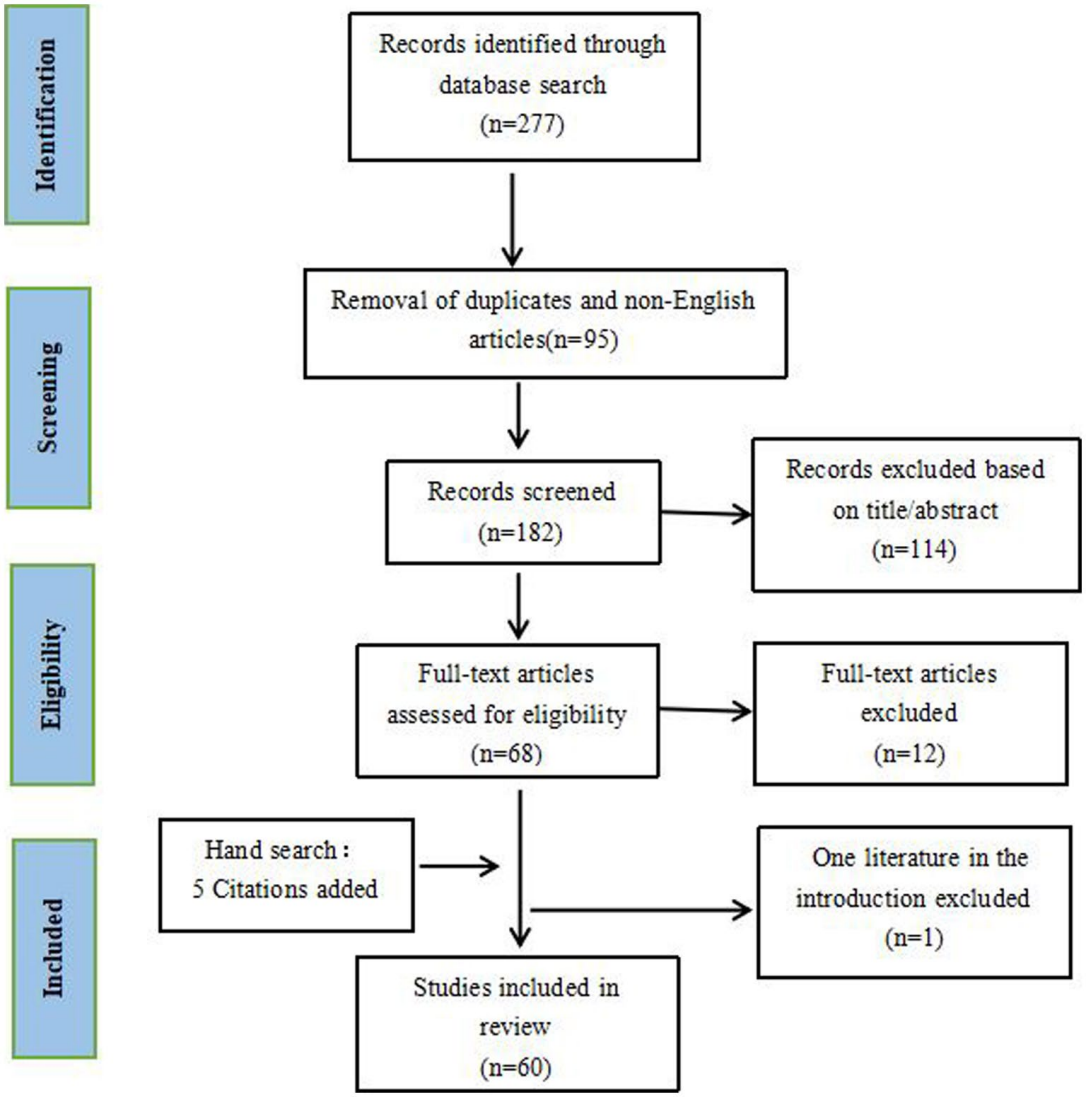
Flowchart demonstrating the schematic of searching and screening strategy.

A literature search was performed in October 2022 using the PubMed, EMBASE, Scopus and Web of Science databases. Search strategies included blepharoplasty, rhinoplasty, rhytidectomy, breast augmentation, breast reduction, mastopexy, abdominoplasty, and liposuction, combined with key subject terms: nerve injury, pain, neuroma, neuropathy, hyperalgesia, paresthesia, and allodynia. Literature was restricted to aesthetic surgeries, ranged from January 1950 to October 2022, and all articles except narrative reviews, editorials, commentaries, and letters were considered. Additional studies were included by reviewing the reference lists of included literature and referencing relevant articles based on search results. Inclusion and exclusion criteria were established before conducting the search, as shown in Table 1. A total of 277 citations were initially identified, 95 of which were eliminated as duplicates or non-English. After evaluating the titles and abstracts according to the inclusion and exclusion criteria (Table 1), 114 articles were excluded as irrelevant studies. Literature screening and evaluation were independently done. Discrepancies were resolved by the two authors through discussion. Hand searching added 5 articles that met our criteria. Furthermore, 12 studies were excluded because the full text was unavailable. Ultimately, a total of 60 studies published between January 1976 and October 2022 were finally included in this review (Figure 2). The quality of the included studies was screened through assessing using the American Society of Plastic Surgeons, Scales for Rating Levels of Evidence (7). The search and screening were done independently by QC, PL, and QZ, and the discrepancies were resolved by communication and discussion.

**Table 1:** Inclusion and exclusion criteria for the literature review.

Inclusion criteria	Exclusion criteria
English language	Non-English language
Study type: randomized controlled trials; cohorts; case series; case reports; cross-sectional studies; meta-analyses; systematic reviews	Study type: narrative reviews; editorials; commentaries; letters
Procedure was blepharoplasty, rhinoplasty, rhytidectomy, breast augmentation, breast reduction, mastopexy, abdominoplasty, and liposuction	Procedure was not blepharoplasty, rhinoplasty, rhytidectomy, breast augmentation, breast reduction, mastopexy, abdominoplasty, and liposuction
Procedure was cosmetic	Procedure was not cosmetic
Procedure was surgical	Procedure was not surgical
Complications included nerve injury, pain, neuroma, neuropathy, hyperalgesia, paresthesia, and allodynia	Complications did not include nerve injury, pain, neuroma, neuropathy, hyperalgesia, paresthesia, and allodynia

## Results

### Blepharoplasty

Blepharoplasty is currently the most popular aesthetic procedure, which improves the appearance of one’s upper eyelids, lower eyelids, or both (8, 9). However, upper blepharoplasty can cause injury to the supraorbital nerve or supratrochlear nerve, while lower blepharoplasty may damage the infraorbital nerve (3). It was reported in several papers that the combination of an infra-brow and a supra-brow resulted in 1.8% (8/432) (10), and 0.8% (5/564) (11) incidence of transient forehead numbness, which gradually subsides within six months with no documented facial nerve injuries. Booth et al. (12) demonstrated the incidence of altered forehead sensation was 74% (32/43 brows), while Lee et al. (13) encountered no such case (14, 15). Turin et al. (16) reported two cases of temporary nerve palsy of the frontal branch of the facial nerve, which resolved completely without permanent nerve damage (150 cases). It is noteworthy that multiple studies (13, 14, 17) have demonstrated the need for careful surgical skills at the supraorbital nerve notch(SON) and glabellar area to avoid damaging the supratrochlear and supraorbital nerves. According to one study (18), the deep branch of the supraorbital nerve traverses the periosteum. It passes between the periosteum and the pericranium towards the top of the head, providing sensation to the frontoparietal scalp. Researchers (11) demonstrated that damage to the supraorbital nerve’s deep branch causes most distress, paresthesia, and numbness following brow lifts, while the superficial branch is more likely to be injured because it penetrates the frontalis muscle to provide sensation to the forehead and anterior region of the scalp. During dissection and fixation, care must be taken to prevent damage to the superficial branch.

Lower eyelid blepharoplasty has a complex anatomy that can lead to injury to the infraorbital nerve (19). In a previous study (20), six patients developed numbness (injury to the infraorbital nerve) following 34 lower eyelid blepharoplasties (17.65%). Five of them recovered within three months; the other recovered within six months after surgery. In another retrospective study (21), 184 consecutive patients received transconjunctival orbital fat grafts without any numbness or nerve injury noted. The levator labii superioris muscle, which overlies the infraorbital nerve, remains intact during surgery as a floor of the premaxillary space (22), so there should be no damage to the nerve. Consequently, to reduce the incidence of injury to the infraorbital nerve (21, 23), the authors suggested that the surrounding soft tissues should be dissected along the sides above the anterior periosteum near the infraorbital anterior portal. To reveal the inferior margin of the orbit, the soft tissues should be pulled away with a cold light retractor.

In the literature cited above, it has been reported that the transient injury incidence of the supraorbital nerve or supratrochlear nerve injury was, on average, 4.0% (45/1119), whereas the frontal branch of the facial nerve was 1.3% (2/150), and the infraorbital nerve was 2.8% (6/218), and no permanent nerve injury has been reported. Therefore, in blepharoplasty, the most frequently injured nerve was the supraorbital nerve or supratrochlear nerve injury.

### Rhinoplasty

Cosmetic rhinoplasty is a popular cosmetic procedure performed globally that encompasses several procedures (24), including alteration of the nasal dorsum, the radix, the nasal tip, and the nasal base, septoplasty, and premaxillary augmentation. Various grafts and allografts are available for cosmetic rhinoplasty, including cartilage, bone, expanded polytetrafluoroethylene, silicone, porous polyethylene, and calcium hydroxyapatite (25). Patients may experience some degree of postoperative olfactory dysfunction following nasal surgery (26). Therefore, any type of nasal surgery, including cosmetic rhinoplasty, may deteriorate olfactory function (27, 28), even without directly damaging the nasal mucosa or the olfactory nerve (29). It should be noted that patients who undertake rhinoplasty need to participate in the Smell Identification Test to evaluate their olfactory function (30–32). Anosmia is an olfactory deficiency diagnosed using the SIT score < 20. Normosmia was referred to as having a SIT score > 35. Hyposmia is termed a composite test score of 20–35.

Furthermore, Shemshadi et al. (33) demonstrated that some degrees of olfactory dysfunction could occur following rhinoplasty, 87.5% (57/65) at postoperative week one. As a result of several studies investigating the effect of open aesthetic rhinoplasty on olfaction, 10 patients (29.5%) (26), 8 patients (10.8%) (34), and 7 patients (10.2%) (35) developed olfaction dysfunction. In particular, Brinner et al. (36) reported that 24% of normal patients (a total of 184 subjects) believed they had impaired sense of smell due to surgery. Conversely, some patients already had an impaired sense of smell before surgery without being unaware of it (36).

The olfactory bulb is mostly vulnerable to damage in the cosmetic rhinoplasty (33, 35, 36). Studies above reported an average incidence of postoperative olfactory dysfunction of 31.8% (26, 27, 33, 34). The patients mostly recovered their baseline olfactory function within six weeks to six months despite initially reducing their function (33, 34, 36).

### Rhytidectomy

A rhytidectomy or facelift consists of a forehead lift, a mid-face lift, and a lower facelift, and most patients undergo two or three procedures. Mitz and Peyronie’s description of the superficial musculoaponeurotic system (SMAS) (37) led to a new epoch in rhytidectomy. Injuries to the facial nerve are among the most severe complications of rhytidectomy. A retrospective study conducted by Baker et al. (38) demonstrated that in a summary of 7,068 cases, the incidence of facial nerve injury was reported to be 0.7% (range 0.4% to 2.6%), and the incidence of permanent injury was 0.1%.

In a recent meta-analysis of the incidence of complications with different facial slimming techniques (39), the permanent injury rate of the facial nerve was reported from 0% to 0.08% for all types of SMAS manipulation techniques. Furthermore, they found that temporary facial nerve injury was reported in SMASectomy/imbrication (0.84%, *n* = 3,454), SMAS flap (0.79%, *n* = 17,247), SMAS plication (0.69%, *n* = 5,081), deep plane (0.69%, *n* = 1,597), and composite (1.52%, *n* = 858), and high lateral SMAS (1.85%, *n* = 1,300). Permanent facial nerve damage occurred in the high lateral SMAS (0.08%, *n* = 1,300) and the SMAS flap (0.04%, *n* = 14,253), and no perpetual facial nerve injuries were covered for the deep plane (*n* = 1795), SMAS plication (*n* = 5,638), SMAS imbrication (*n* = 3,254), and composite (*n* = 727).

Researchers (40) found a provisional facial nerve injury rate of 7.1% (primary surgery) and 2.2% (secondary surgery), with both primary and secondary facelifts using lamellar SMAS techniques (41).

Multiple studies (40–42) have explained the reasons for facial nerve injury, including suture placement, transection, electrocauterization, excessive traction and stretching, crushing injuries caused by surgical instruments, distorted anatomy, and adhesion from a previous procedure. In some studies (43, 44), researchers have suggested that facial nerve function generally recovers three to six months after an injury due to multiple interconnections and intermingling of the facial nerve branches (45).

Anatomical studies (41, 44, 46, 47) have further confirmed that the frontal branches are separate from the SMAS and contained within a distinct layer.

Therefore, according to the studies mentioned above, the incidence of facial nerve injury is nearly 1.0% (temporary) and 0.03% (permanent). The marginal mandibular branch and the cervical branch are most vulnerable to be injured. It is also beneficial to have a good understanding of the location of facial nerve branches, the retaining ligaments, and the soft-tissue plane of the face to decrease the incidence of facial nerve injury.

### Breast surgeries

Aesthetic breast surgeries usually include augmentation, mastopexy, and reduction mammaplasties, with breast augmentation being the most frequently performed procedure (48). As part of a retrospective study by Broyles et al., all cases of augmentation, mastopexy, and reduction who complained of postoperative chronic pain failed to respond to physical or drug therapy (49). Surgical repair was then projected. Anesthesia blockade of suspected injured nerves was found to be effective prior to surgery. One or more intercostal nerves were found to be injured and resected and implanted into adjacent muscles. Except for the patient who underwent augmentation, both mastopexy and reduction patients responded well to therapy (49).

Brown et al. conducted a prospective study to examine objective sensory changes using a Semmes Weinstein monofilament following subfascial breast augmentation (50). According to the study, the greater the initial breast volume, the greater the sensory loss at 12 weeks in the nipple-areolar complexes. The calculated ratio was 102% to 4%. Helmy et al.’s study examined nipple-areolar complex sensitivity changes, showing a low incidence, which rebounded one year later to 1.66% (51). A peri-areolar approach tends to have fewer sensation changes.

Another retrospective study conducted by Ducic et al. (5, 52) demonstrated that 74% of the patients that presented with pain in the surgical scar had concomitant injuries of intercostal nerves (2nd to 7th) due to mechanical trauma of sharp dissection or compressive scar entrapment. As a result of blunt tissue dissection, 7% suffered from traction-stretch neuropathy. Furthermore, the lateral zone could be the most damaged area (79%). Specifically, augmentations through peri-areolar incisions tended to injure intercostal nerves (3rd to 4th) in the central zone, whereas the inframammary approach mostly affected intercostal nerves (5th to 6th) in the inferior zone, and for transaxillary incisions, the second intercostal nerve in the lateral zone was the most injured. Intercostal nerves (3rd to 5th) in the central, inferior, and lateral zones were involved in breast reduction, and intercostal nerves (3rd to 4th), usually in the central zone, were affected by mastopexy. Their recommendations included physical therapy, avoiding compressive garments, and corresponding oral medications in the initial stages of injured nerve symptoms. If the symptoms persist beyond three to six months, surgical treatment with selective neuroma excision and proximal end implantation is advised. The effectiveness of the recommended treatment has not been systematically tested.

During cosmetic breast surgeries, the intercostal cutaneous nerves are most vulnerable to damage (8.86% to 10.01%). It is estimated that 13.57% of total injuries and 1.66% of permanent injuries occur on average during cosmetic breast surgeries (5, 51). It is interesting to note that the injury not only results from sharp or blunt dissection but is also determined by the volume of prosthesis implanted in augmentation mammaplasties.

### Abdominoplasty

Aesthetic abdominoplasty is a cosmetic surgery that removes excess skin and fat, tightening the muscles and fascia to make the abdomen thinner and firmer (53, 54). A retrospective study by Chatel et al. evaluated persistent postsurgical pain in 67 patients who underwent abdominoplasty using a visual analog pain scale (VAS) with the Douleur Neuropathique 4 (DN4) questionnaire (55), of these patients, 19.4% (13) reported neuropathic pain accompanied by hypoesthesia or hyperesthesia. The identified risk factors were acute postoperative pain, history of bariatric surgery, prolonged hospitalization, depressive status, substantial stress, and significant postoperative complications. Though specific nerve injuries were not discussed, the authors emphasized the importance of preemptive approaches and early postoperative diagnosis.

Another retrospective study done by Uchelen et al. (56) reviewed 86 patients who had underwent abdominoplasty found that 9 patients (10.5%) were documented with nerve injuries. Among which, 8 patients (9.3%) experienced sensibility disorder of the thigh. Special attention to preserve the lateral cutaneous nerves of the thigh was emphasized by the authors.

Pechter et al. described a case of right femoral nerve damage that manifested as the patient being unable to extend her right knee immediately following the performance of abdominoplasty. Her right patellar reflex and sensibility in the anteromedial thigh and medial leg were diminished (57). Two days after surgery, the patient was subjectively better and fully recovered without any surgical complications or neurologic deficits. Considering her rapid recovery, it is likely that she suffered a mild neuropraxic injury or a possible side effect of local anesthetic. Another case reported by Ege et al. (58), who underwent abdominoplasty 3 weeks ago, sustained the anterior abdominal skin burnt resulted from sensory loss. The case reminded us the importance to inform our patients of possible skin injury due to the loss of sensation.

An anatomical study by Chowdhry et al. conducted a surgical recommendation to prevent lateral femoral cutaneous nerve injury in abdominoplasty (59). Based on their dissection of 23 fresh cadaver abdomens, careful dissection in about 4 cm anterior superior iliac spine and preservation of the scarpa fascia near the inguinal ligament can provide a safe strategy to avoid lateral femoral cutaneous nerve injury in abdominoplasty.

According to a review by Ducic et al., specific nerve injury rates were 1.94%, and permanent injury rates were 1.02% after abdominoplasty (6). Among all the patients, the rates of hypoesthesia, chronic pain, and temporary weakness or paralysis were 7.67%, 1.07%, and 0.44%, respectively. The nerves directly injured were the lateral femoral cutaneous nerves (1.36%) and iliohypogastric nerves (0.10%) (60–62). The authors recommended a timely, multidisciplinary treatment to optimize symptoms related to nerve injury, including a rigorous diagnostic examination followed by initial conservative treatment. Medication treatment with painkillers, nerve blocks, or steroid injections are also recommended in the short term. A peripheral nerve surgeon should be consulted if the symptoms persist for over three months.

Based on the studies, the most injured nerve in cosmetic abdominoplasty is the lateral femoral cutaneous nerve. A permanent nerve injury occurs in 1.02% of cases. In abdominoplasty, nerve injuries may be reduced by carefully handling the anterior superior iliac spine and area near the inguinal ligament.

## Discussion

Although cosmetic surgery is on the rise, plastic procedures remain the most impregnable in comparison with burgeoning non-invasive or minimally invasive procedures due to their radical improvements. However, radical changes caused by these invasive operations are the source of iatrogenic complications (63). Although peripheral nerve injuries are rare, they continue to be a serious concern for affected patients and plastic surgeons. Thus, all plastic surgeons should be familiar with the vulnerable nerves for specific surgeries, respectively, to reduce the possible damage as much as possible. This review, though not complete, lists the vulnerable nerves and their symptoms following commonly performed plastic surgeries. The complication was discussed in relation to preventing, diagnosing, and treating it to provide some information for plastic surgeons (Table 2). Raising concerns in a timely manner is essential.

**Table 2:** Weighted rates of nerve complications by cosmetic surgery.

Surgery type	Complication type	Complication rate %	Treatment	Prognosis
Blepharoplasty	Supraorbital nerve or supratrochlear nerve	4.0	Conservative	Fully recovery
	The frontal branch of the facial nerve	1.3	Conservative	Fully recovery
	Infraorbital nerve	2.8	Conservative	Fully recovery
	Permanant nerve injury	N/A	N/A	N/A
Rhinoplasty	Olfactory dysfunction	31.8	Conservative	Fully recovery
	Permanant nerve injury	N/A	N/A	N/A
Rhytidectomy	Facial nerve injury	1.0	Conservative	Fully recovery
	Permanant nerve injury	0.03	Conservative & surgery	Partially recovery
Breast surgeries	Intercostal cutaneous nerve	8.86 to 10.01	Conservative	Fully recovery
	Long thoracic nerve	0.01	Conservative	Fully recovery
	Permanant nerve injury	1.66	Conservative	Partially recovery
Abdominoplasty	The lateral femoral cutaneous	1.36	Conservative	Fully recovery
	Iliohypogastric	0.1	Conservative	Fully recovery
	Permanant nerve injury	1.02	Conservative & surgery	Partially recovery

Conservative treatment: pain medications, nerve blocks, or steroid injections.

### Treatment algorithm

**Figure 3:**
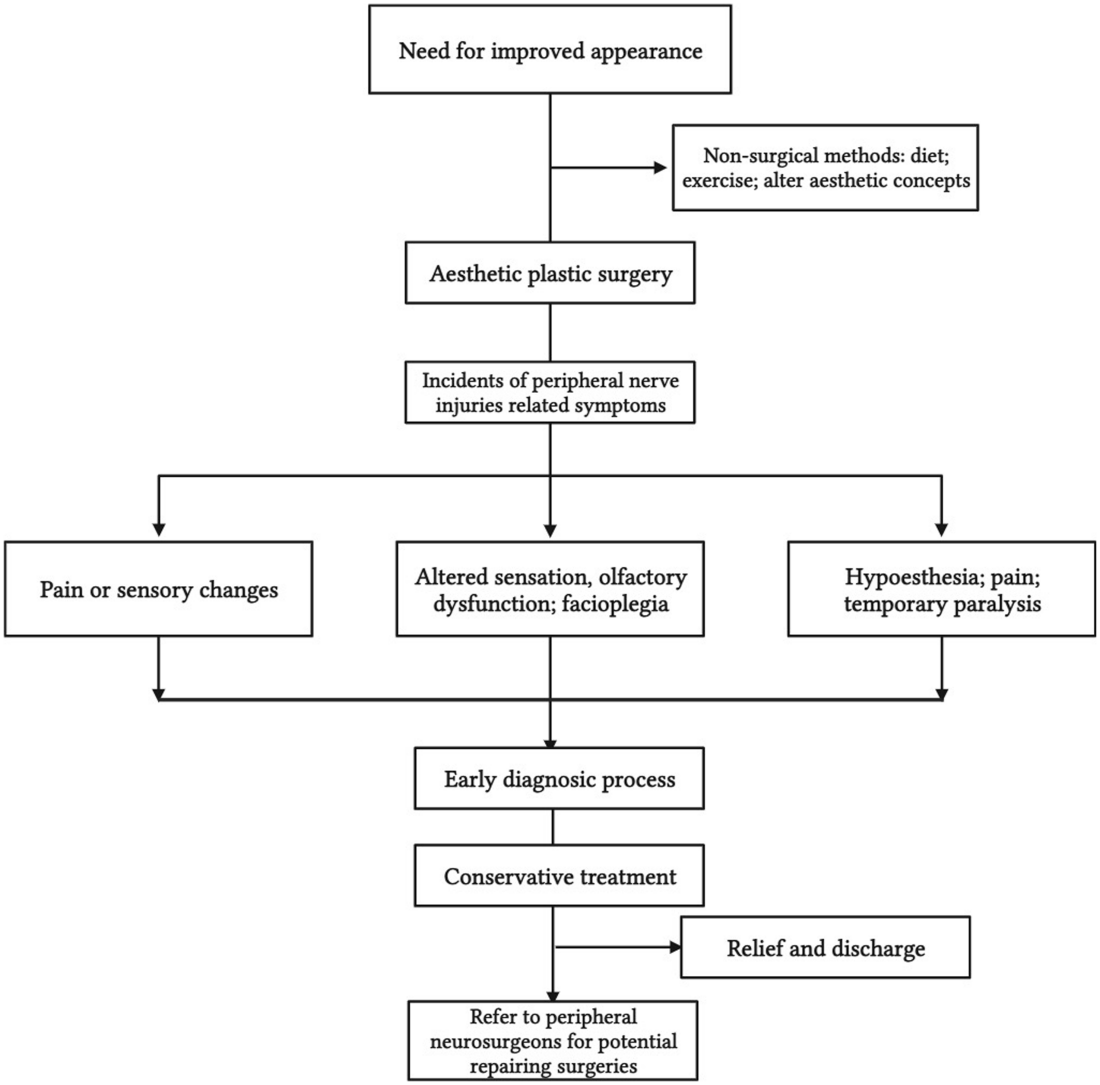
A detailed treatment algorithm relating complications of nerve injuries.

There are several challenges associated with nerve injuries, both in terms of diagnosis and therapy. Based on the opinions of the authors of included studies, we developed a recommended treatment algorithm to assist fellow surgeons with various problems (Figure 3).

Before surgeries, patients should be informed of the potential symptoms of nerve injuries, including chronic pain, hyperesthesia, hypoesthesia, and possible numbness. Mastering the related anatomy and careful dissection are necessary for a better outcome at surgery. Prevention is better than treating injuries after they have occurred.

Although some pain and paresthesia are expected to be normal in the early postoperative period, symptoms over three months can indicate nerve injuries. Early diagnosis and treatment are crucial (64) because postponed or improper treatment can result in irreversible consequences or even additional morbidities (65, 66). In addition to a comprehensive physical examination, common complications such as infection, seroma, hematoma, or fluid collection, together with other probable causes, for example, hernia, endometriosis, or abdominal visceral diseases, should be excluded. A Tinel’s sign or relief of tenderness after the targeted injection of anesthetic nerve blocks proximal to the focal point is a strong indication of nerve injury (3). Providing the diagnosis to patients validates the cause of their symptoms and reduces their emotional stress (67).

Initially, conservative modalities, such as scar massage, desensitization, and sensory reeducation, are advised for symptoms associated with some nerve injuries that can self-resolve over time. Furthermore, medication treatment that includes pain relievers, tricyclic antidepressants, anticonvulsants, steroid injections, or nerve blocks may also be helpful (6). Besides, standardized diet, nutrition, and supplementation recommendations and protocols may be of great importance for better nerve regeneration and functional recovery (68). If no improvement is seen over the first three months, referring to a peripheral nerve surgeon for possible surgical treatment, which involves exploring the suspected injured nerves with corresponding management, such as neurolysis, neurectomy, and implantation, is necessary (69, 70). This algorithmic approach generally eliminates most symptoms, but prevention remains the best treatment.

### Limitations and future

While this narrative review has provided insights into peripheral nerve injuries following cosmetic surgeries, there are several limitations to consider: 1. Limited Sample Size: the rarity of peripheral nerve injuries in the context of cosmetic surgeries makes it challenging to gather a large sample size for systematic analysis. 2. Potential Bias: the existing literature on peripheral nerve injuries may exhibit a bias towards highlighting severe or exceptional cases. Moreover, it appears that the majority of documented peripheral nerve injuries have a sensory component. This bias could be attributed to the influence of personal emotions, potentially impacting the objectivity of the reported results. 3. Heterogeneity of Data: the studies included in this review vary in terms of study design, patient demographics, surgical techniques, and follow-up periods. This heterogeneity can make it difficult to draw uniform conclusions and may introduce confounding variables. 4. Lack of Long-term Follow-up: many of the studies reviewed primarily focus on the immediate postoperative period and short-term outcomes. Long-term follow-up data on the persistence of nerve injuries and their impact on patients’ quality of life are often lacking. 5. Variable Reporting Standards: the reporting of peripheral nerve injuries across different studies may lack uniformity, making it challenging to compare and compile data accurately.

To address these limitations and further advance our understanding of peripheral nerve injuries following cosmetic surgeries, future research should consider the following aspects: 1. Prospective Studies: conducting large-scale, multicenter prospective studies specifically focused on peripheral nerve injuries in cosmetic surgeries would provide more robust data and reduce selection bias. 2. Standardized Reporting: the development of standardized criteria for reporting peripheral nerve injuries and their outcomes is essential. This would facilitate better comparisons between studies and enhance the accuracy of meta-analyses. 3. Long-term Follow-up: longitudinal studies with extended follow-up periods are necessary to assess the long-term consequences of peripheral nerve injuries and the effectiveness of various treatment modalities on patients’ quality of life. 4. Advancements in Preventative Techniques: research should continue to explore innovative techniques and technologies aimed at reducing the occurrence of iatrogenic peripheral nerve injuries during cosmetic surgeries. This may include the use of advanced imaging, surgical tools, and training methodologies. 5. Patient Education: emphasizing patient education about the potential risks and complications associated with cosmetic surgeries, including peripheral nerve injuries, can help patients recognize early symptoms and contribute to better prognosis. 6. Collaboration: collaboration between plastic surgeons, neurologists, and other medical specialties can lead to a more comprehensive understanding of peripheral nerve injuries, improved prevention strategies, and better treatment options.

In the realm of cosmetic surgeries, it is paramount that we emphasize the utmost care and precision to safeguard against possible nerve injuries. To minimize the risk of such injuries, practitioners should carefully assess the patient’s anatomy, taking into consideration any potential variations or anomalies. During surgery, gentle tissue handling and a thorough understanding of the nerves’ pathways are important to avoid inadvertent damage. Adequate lighting, magnification, and proper instruments can aid in preventing unintended injuries. Vigilance and meticulous attention to detail are essential. Moreover, post-operative monitoring and communication with patients are equally crucial to promptly identify and address any signs of nerve impairment. By adhering to these principles and prioritizing nerve preservation, we can improve the overall safety of cosmetic surgical procedures.

## Conclusion

Peripheral nerve injuries are rare but deserves special attention. Albeit the postoperative algorithm approaches prove effective, the waiting and treating processes can be lengthy and painful. Preventive measures are always preferable to postoperative remedies. Good mastery of surgical anatomy and meticulous dissection are the best weapons of prevention.

## Author contributions

QC: Conceptualization, Formal analysis, Methodology, Funding acquisition, Software, Writing - original draft. PL: Data curation, Formal analysis, Investigation, Methodology, Writing - original draft, review & editing. QZ: Project administration, Resources, Visualization, Writing - original draft. TT: Methodology, Supervision, Validation, Writing – review & editing. HL: Conceptualization, Formal analysis, Funding acquisition, Supervision, Writing – review & editing. WZ: Formal analysis, Funding acquisition, Resources, Supervision, Validation, Writing – review & editing.
